# Characterization and complete genome sequence analysis of a newly isolatedphage against Vibrio parahaemolyticus from sick shrimp in Qingdao, China

**DOI:** 10.1371/journal.pone.0266683

**Published:** 2022-05-04

**Authors:** Fengjuan Tian, Jing Li, Yunjia Hu, Feiyang Zhao, Huiying Ren, Qiang Pan, Amina Nazir, Fei Li, Yigang Tong

**Affiliations:** 1 College of Life Science and Technology, Beijing University of Chemical Technology, Beijing, China; 2 Qingdao Phagepharm Bio-tech Co., Ltd, Shandong, China; 3 Institute of Animal Science and Veterinary Medicine, Shandong Academy of Agricultural Sciences, Shandong Province, China; 4 Center for Clinical Laboratory, The Affiliated Taian City Central Hospital of Qingdao University, Taian, Shandong, China; University of Patras, GREECE

## Abstract

Foodborne diseases have become a serious havoc, where antimicrobial resistance is throwing significant challenges on daily basis. With the increase of drug-resistant bacteria and food-borne infection associated with *Vibrio parahaemolyticus*, new and effective strategies were needed to control the emergence of vibriosis. Lytic bacteriophages come up as a promising way to resist the pathogenic population in various applications. In this study, a *V*. *parahaemolyticus* specific phage vB_VpS_PG28 was isolated from sewage in the seafood market. Results showed vB_VpS_PG28, is strictly a lytic bacteriophage and has a relatively large burst size of 103 plaque-forming units per infected cell. Comparative genomic and bioinformatic analyses proved that vB_VpS_PG28 is a new bacteriophage that had a homologous relation with *Vibrio* phages of family Siphoviridae, especially with phage VH2_2019, but transmission electron microscopy of vB_VpS_PG28 morphology characterized its morphology is similar to that of Myoviridae family. *In silico* analysis indicated that the vB_VpS_PG28 genome consists of 82712 bp (48.08% GC content) encoding 114 putative ORFs without tRNA,and any gene associated with resistance or virulence factors has not been found. The bacteriophage in the present study has shown significant outcomes in order to control bacterial growth under *in vitro* conditions. Thus, we are suggesting a beneficiary agent against foodborne pathogens. Further, to ensure the safe usage of phage oral toxicity testing is recommended.

## 1 Introduction

With the passage of time morbidity and mortality rate has increased due to foodborne diseases all around the world [1]. Moreover, sickness threats due to foodborne pathogens are aggravated by trading of food products globally. Infectious outbreaks related to foodborne pathogens significantly threatens human health and economies [2]. *Vibrio parahaemolyticus* (*V*. *parahaemolyticus*) is a gram-negative motile (halophilic) bacterium residing in marine (fish, shellfish) and estuarine environments all over the world [3–7]. Most gastroenteritis-related diseases are caused by this foodborne pathogen [8]. Contaminated raw or undercooked seafood are the main reasons of foodborne infections. The presence of this bacteria in seafood is a risk for public health, especially in the regions where shellfish is used as raw food. Antibiotics are being used in order to control the *V*. *parahaemolyticus* in aquaculture production. However, frequent use of antibiotics leads towards multiple-antibiotic resistances, mostly against Ampicillin and Streptomycin [9]. Environmental strains of *V*. *parahaemolyticus* are non-pathogenic strains. However, clinical strains yield thermostable direct hemolysin (TDH), and other virulence factors [10, 11]. Therefore, alternative strategies are needed to prevent and control *V*. *parahaemolyticus* infections.

Bacteriophages (phages) are viruses that can specifically infect and kill the bacteria [12]. They are natural antibacterial agents and reproduce only in their specific host bacterium. Due to this specificity, phage lysates are widely used as effective antibacterial agents for the treatment of bacterial diseases [13, 14]. Phage therapy is an environment-friendly pathway to overcoming the drug-resistant pathogenic bacteria due to its specific properties of lysing bacterial cells [15]. At present, phage cocktail therapies and combinations of phage and antibiotics are used for the treatment of patients with serious bacterial infections. Phage therapies are successfully employed in many countries including Georgia,China, USA, Poland, and France [16–19]. Furthermore, phages are also used in other fields including agriculture, poultry, sewage and food production for the control of bacterial diseases. However, before determining the potential of bacteriophages as antimicrobial agents it is essential to get better insight of phage biology [13, 20–23].

Bacteriophages having lytic life cycle are potential candidates for biocontrol. It’s not only the morphology but also the genetic makeup which helps to determine the potential of these phages against pathogenic bacteria because of their virulent genes [24]. Therefore it is necessary to sequence the complete genome of these bacteriophages to acquire the knowledge about their function as biocontrol agent [25, 26].

This study aims to isolate and characterize the novel polyvalent biocontrol agent (vB_VpS_PG28) with a broad-spectrum activity against an MDR (multidrug resistance) strains of *V*. *parahaemolyticus*. Detailed genomic and biological properties of phage vB_VpS_PG28 were analyzed. The outcome may result to get potent biocontrol phages thathelp to combat with pathogenic bacteria.

## 2 Materials and methods

### 2.1 Bacterial strain and culture conditions

The host bacteria were isolated from diseased shrimp in Qingdao, China and initially cultured on 2216E medium (Solarbio, Beijing). The isolated strain was confirmed by amplifying a 1463-bp fragment of the 16S rRNA gene with the universal primer pair27F:5’-AGAGTTTGATCCTGGCTCAG-3’ 1492R:5’-GGTTACCTTGTTACGACTT-3’. Then, the Sanger sequencing was performed to obtain the sequence of the PCR product. The resulting sequence was compared against the GenBank database using BLASTn and the best match result calculated by BLAST was used to determine the identification of the bacteria. 30% glycerol was used to store the strain at −80°C and routinely grown in liquid 2216E medium (Solarbio, Beijing) at 37°C overnight.

### 2.2 Phage isolation and purification

Phage vB_VpS_PG28 was isolated from sewage at the seafood market in Qingdao. The *V*. *parahaemolyticus* isolated from diseased shrimp was used as host bacteria for phage isolation. Firstly, the sewage from the seafood market was centrifuged and the supernatant was filtered through a 0.22μm syringe filter. Then, it was enriched, mixed with the cultured bacterial liquid and incubated at 37°C for 6h. After high-speed centrifugation, the supernatant was filtered through a 0.22μm syringe filter. 100 μL filtrate was mixed with 500μL bacterial liquid, and the mixture was presented on the double layer agar plate. Specific phages were isolated by double agar plate method [27], and purified after the appearance of bacterial plaque. An aseptic inoculation ring was used to pick the single plaque and eluted it into the host bacterial culture, and the mixture was again plated on double layer agar plate. To obtain the purified phages, this experiment was repeated three times. The purified phages were counted and the plaque-forming units (PFU/mL) in each dilution were recorded. Purified phages were stored in 2216E fluid medium at 4°C. Stock cultures were stored in 2216E broth supplemented with 50% glycerol at -80°C.

### 2.3 Optimal multiplicity of infection (MOI)

The phage and host bacteria were mixed according to MOI of 1, 0.1, 0.01, 0.001 and 0.0001, respectively, and cultured at 37°C for 6h. After high-speed centrifugation (12000×g for 2 mins), supernatant was filtered through a 0.22μm syringe filter to obtain phages. Phage titer was determined using the double agar layer method. The experiment was performed in triplicates.

### 2.4 Thermal and pH stability

The thermal and pH stabilities of vB_VpS_PG28 were evaluated under the optimal MOI conditions as previously described with some modifications [28, 29]. For thermostability test, the isolated phages were incubated at different temperatures (40°C, 50°C, 60°C and 70°C) with the pH condition of 7.4to test whether the phage could tolerate high temperatures, and aliquots (100 μL) were collected at 20, 40, and 60 mins during the incubation, respectively. In addition, the phage content at 0 min represents the results of a test at room temperature. For pH stability assay, PBS was used for the incubation of the isolated phages over a pH range (2–13, adjusted using NaOH or HCl) at 37°C for 1h. Double agar layer method was used to determine the phage titer. All experiments were performed at least three times.

### 2.5 Electron microscopy

The purified phage sample was obtained by centrifugation at 12000×g for 2 mins. The supernatant was filtered through a 0.22μm filter. Ten microliters of purified phage solution were poured onto a copper grid and rested it for 1 min. Then, for staining the samples, 2% phosphotungstic acid was used and extra solution was washed out. Then waiting until grids were air dried and observed with a TalosL120C transmission electron microscope (FEI, Hillsboro, OR, USA) set at 120 kV to obtain the phage morphology.

### 2.6 Host range analysis

Host range analysis was performed by testing phage vB_VpS_PG28 against 14 strains of *Vibrio* species. Double agar plate method was used to check the host specificity of phage by spotting a 10μL drop of phage lysate on the plate surface, followed by overnight incubation at 37°C. Subsequently, phage activity was examined visually by clearance zones represented bacterial cell lysis.

### 2.7 One-step growth curve

Phage latent period and phage burst size were determined as described in [30, 31] with some modifications. Under the optimal MOI conditions, the phages and host bacteria were mixed, incubated at 37°C for 5 mins, centrifuged at 12000 rpm for 30 s, and the supernatant was discarded. The precipitate was washed with 2216E medium, centrifuged at 12000 rpm for 1 min, and repeated three times to remove the unabsorbed host bacteriophages. 20 ml 2216E liquid medium was added and cultured at 37°C. Samples were taken every 10 mins from 0 moment and centrifuged at 12000 rpm for 2 mins. The supernatant was collected, and the titer of phage was determined. The one-step growth curve was drawn by taking the infection time and phage titer (PFU/ml). Furthermore, the incubation and lysis period of the phage were attained, and the amount of lysate was calculated. All experiments were repeated at least for three times.

### 2.8 DNA extraction

Phage genomic DNA was extracted by the phenol-chloroform method [32]. For DNA extraction, 1mL of phage lysate was treated with 1 μg/mL of RNase A and with DNase I similar quantity at 37°C overnight, followed by incubation at 80°C for 15 mins to deactivate DNase I and RNase A. Then, Proteinase K (50 μg/mL) treatment was given to purified phages at 56°C for 30 mins, supplemented with SDS (0.5%) and EDTA (20 mM). An equal volume mixture of phenol/chloroform/isoamyl alcohol (25:24:1) was added, mixed and centrifugation was done at 4°C for 10 minutesat 12,000 g. The supernatant was transferred and mixed with an equal volume of chloroform and centrifuged at 10,000 g for 5 mins. The supernatant was transferred and mixed with 400μl isoamyl and stored at -20°C for more than 1h. Then centrifugation was done, to wash away the organic solution from DNA, it was treated with 70% ethanol and DNA pellet was air dried. Dried DNA was then dissolved in 30μl of nuclease free water and stored at -20°C. Spectrophotometer was used at 260nm wavelength to measure the DNA concentration.

### 2.9 Genome sequencing

The product of genome extraction was used to construct a 600 bp insert length library using the NEBNext® Ultra™ II DNA Library Prep Kit for Illumina. Illumina Miseq (San Diego, Ca, USA) was used for high-throughput sequencing. De novo assembly with 392,358 trimmed reads (97.88% of raw reads) was performed using SPAdes v3.13.0.

### 2.10 Genome sequence analysis and bioinformatics analysis

Packaging mechanism was determined using PhageTerm tool [33]. RAST (http://rast.nmpdr.org/) online annotation server was used to annotate the whole genome of phage. Blastp (http://www.ncbi.nlm.nih.gov/BLAST) against non-redundant protein database was used to further predict the functions of annotated proteins. The virulence determinants and the genes involved in antibiotic resistance were determined using the Virulence Factor Database (VFDB) [34] and ResFinder [35] (https://cge.cbs.dtu.dk/services/ResFinder) respectively. Genes encoding tRNAs were predicted by the tRNAscan-SE program [36] (http://lowelab.ucsc.edu/tRNAsc an-SE/). Circular genome mapping was performed using an in-house python script. VIRIDIC (http://rhea.icbm.uni-oldenburg.de/VIRIDIC/) was used in order to determine the intergenomic similarity and differences of phage vB_VpS_PG28 with other phages [37]. A comparative analysis of the phage genome with its closest relatives was conducted using Easyfigv2.2.3 at the DNA level [38]. And the data was downloaded from NCBI(https://www.ncbi.nlm.nih.gov). Two proteins, terminase large subunit (ORF19) and DNA polymerase (ORF31) were used to construct the phylogenetic trees to infer the evolutionary history of proteins along with target phage vB_VpS_PG28. The homologous sequences of these two proteins were downloaded from NCBI. The Neighbour-Joining (NJ) method in MEGA v7.0 [39] was used to generate the trees with 1000 bootstrap.

## 3 Results and discussion

### 3.1 Bacterial strain

**Fig 1:**
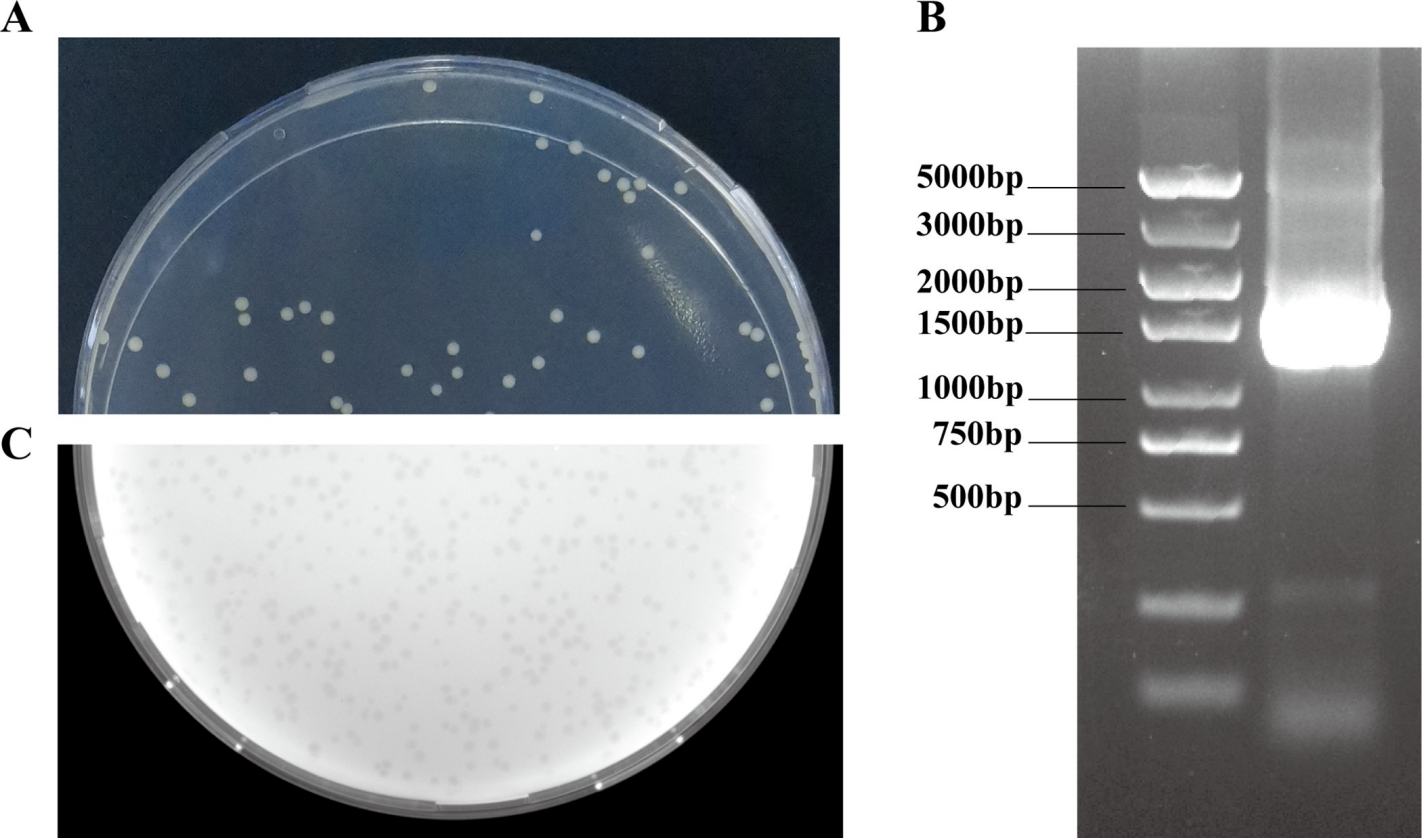
**(A)** The coated plate of *V*. *parahaemolyticus* 6A. **(B)** Gel electrophoresis result of16s PCR of *V*. *parahaemolyticus* 6A. **(C)** The formed plaques of phage vB_VpS_PG28 using *Vibrio parahaemolyticus* 6A as a host strain.

The bacterial strain *V*. *parahaemolyticus* 6A, isolated from diseased shrimp, formed circular and small (*≤*1 mm diameter) off-white translucent colonies **(Fig 1A)**. The bacterial strain used as a host strain in this study was isolated from diseased shrimp in Qingdao, China. 16s rRNA gene sequencing was done to confirm the bacterial host strain as a *V*.*parahaemolyticus* **(Fig 1B)**.

### 3.2 Phage isolation

**Fig 2:**
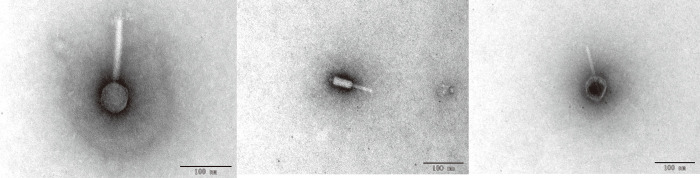
Morphology of the phage vB_VpS_PG28 under transmission electron microscopy.

Phage vB_VpS_PG28 was isolated from sewage at the seafood market in Qingdao. Phage produces clear plaques on double agar 2216E plates after co-culturing with *V*. *parahaemolyticus* 6A **(Fig 1C)**. Plaques formed by phage vB_VpS_PG28 were 1.5 to 2.0 mm in diameter with well-defined boundaries against the *V*. *parahaemolyticus* bacterial host strain. Morphology and plaque size may differ in their measurements according to growth conditions, but it was observed that typical virulent phagesproduce clear plaques. Oppositely, phages those have ability to lysogenize form turbid plaques, substantiated that vB_VpS_PG28 may be initially assessed as a virulent phage. Morphological characteristics of phage vB_VpS_PG28 under the transmission electron microscopy (TEM) indicated that it possessed isometric, icosahedral capsids (approximately 56±3 nm in diameter) and contractile tails **(Fig 2)**. Bacteriophages are classified according to the morphology of their virion characteristics.Variable tail morphology was observed in the vB_VpS_PG28 sample, which is similar to Myoviridae phage.

### 3.3 Host range

The host range of the phage was assessed against *Vibrio* hosts by using spot test. Results showed that phage vB_VpS_PG28 form clear plaques against host bacteria suggesting that phage vB_VpS_PG28is more specific. In addition, it was only susceptible to its host bacteria and *V*. *parahaemolyticus* 2294 within the test range.Itcould not be able to form plaques against other *vibrio* strains **(Table 1)**.

**Table 1:** Lytic activity of vB_VpS_PG28 against tested *V*. *parahaemolyticus* and *V*. *alginolyticus* strains.

Bacterial Strain	Phage Sensitivity^a^
*Vibrio parahaemolyticus* 1420	-
*Vibrio parahaemolyticus* 1639	-
*Vibrio parahaemolyticus* 1652	-
*Vibrio parahaemolyticus* 2216	-
*Vibrio parahaemolyticus* 2286	-
*Vibrio parahaemolyticus* 2287	-
*Vibrio parahaemolyticus* 2290	-
*Vibrio parahaemolyticus* 2294	+
*Vibrio parahaemolyticus* 2300	-
*Vibrio parahaemolyticus* 2305	-
*Vibrio parahaemolyticus* 2310	-
*Vibrio alginolyticus* 2205	-
*Vibrio alginolyticus* 2220	-
*Vibrio alginolyticus* 2230	-

^a^Symbols: (+) clear zones or (−) no plaques after infection of tested bacteria with vB_VpS_PG28bacteriophage.

### 3.4 Biological characteristics of phagevB_VpS_PG28

**Fig 3:**
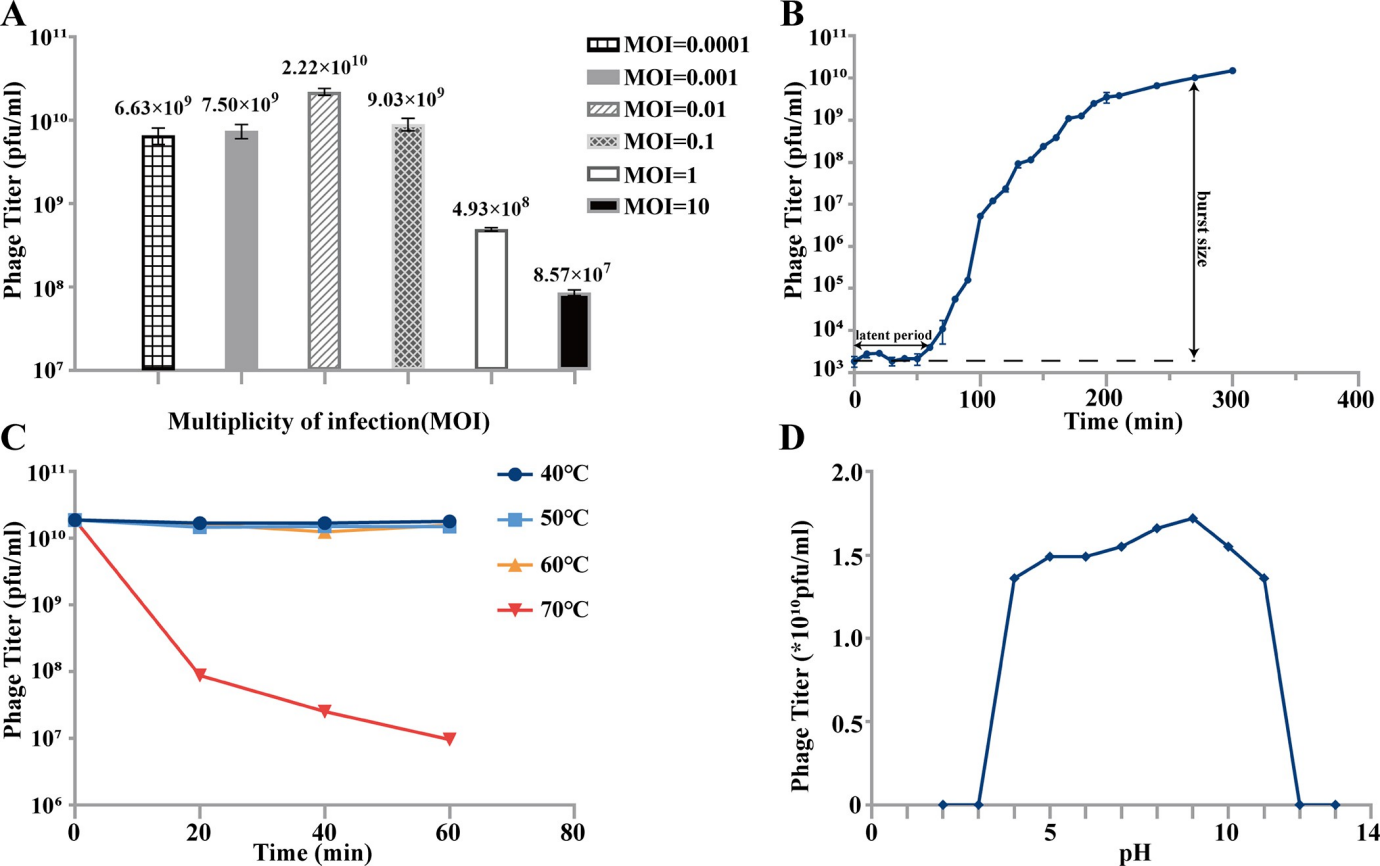
Biological characterization of the phage vB_VpS_PG28. **(A)** The multiplicity of infection (MOI) test of vB_VpS_PG28; **(B)** The one-step growth curve of phage vB_VpS_PG28, and data points show phage titers measured at ten-minute intervals; **(C)** Thermostability curve of vB_VpS_PG28, and data points are phage titers measured after incubating the phage at different temperatures for 20, 40 and 60 minutes respectively. **(D)** pH stability of vB_VpS_PG28, and data points are phage titers measured after incubation of phage at different pH for 1h. All assays were performed in triplicate.

MOI results indicate that the optimal multiplicity of infection is 0.01, when mixing10^6^ phages with 10^8^ cells **(Fig 3A)**. The one-step growth experiment was performed to investigate the growth parameters by observing phage growth cycle. The result exhibited that the latent period and burst period of phage vB_VpS_PG28 were 60 mins and 210 mins, respectively, and an average burst size was about 103 plaque forming units (PFUs)/infected cell **(Fig 3B)**. Thermal and pH tolerance represented the range of application for the phage. The thermal stability test showed that vB_VpS_PG28 was stable below 60°C and the stability decreased gradually at 70°C **(Fig 3C)**. In addition, phage vB_VpS_PG28 remained active over a wide pH range (pH 4–11), which suggests that phage vB_VpS_PG28 could be applied in harsh environment **(Fig 3D)**.

### 3.5 General features of phagevB_VpS_PG28

**Fig 4:**
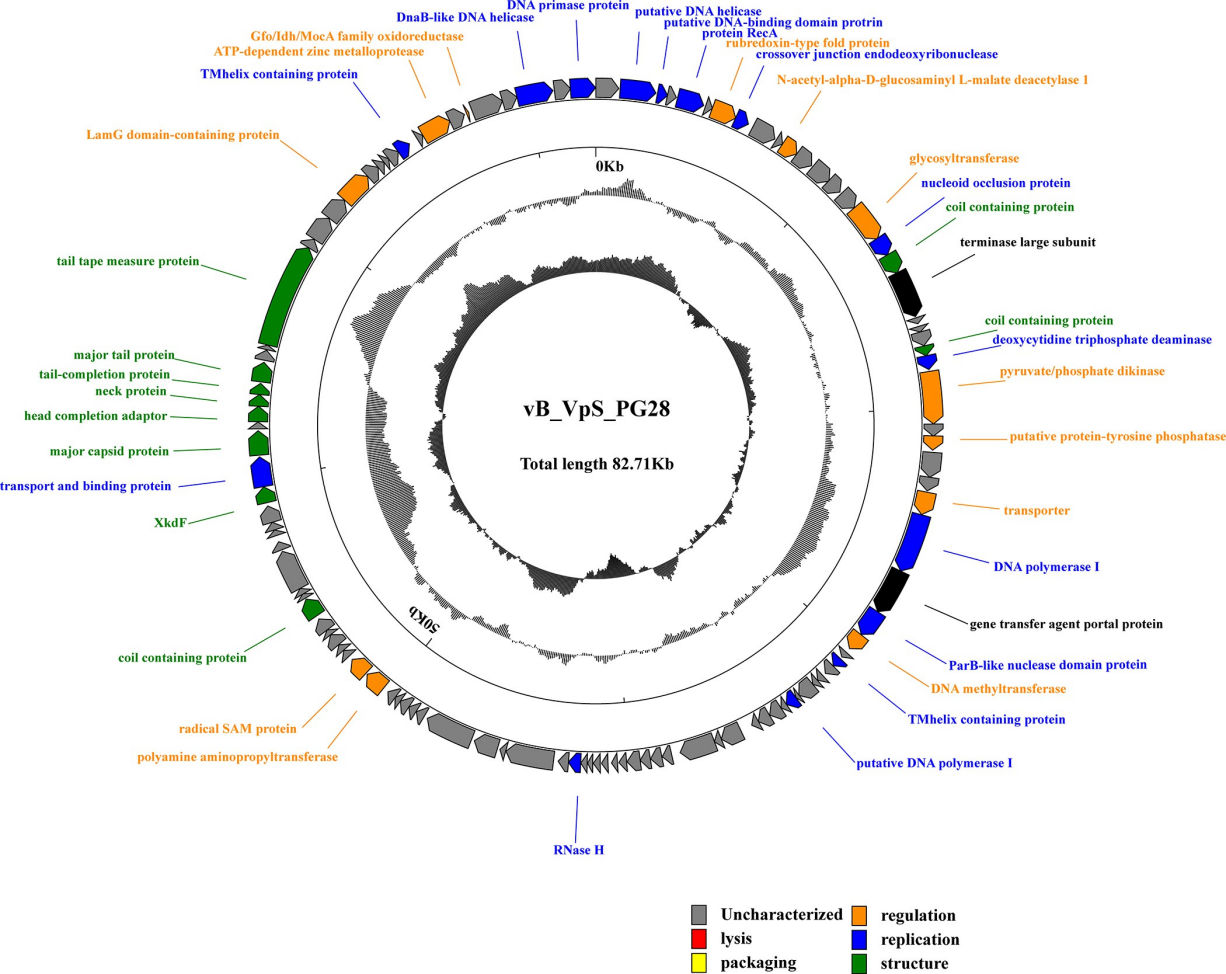
Circular map of the bacteriophage vB_VpS_PG28 genome. The innermost circle represents the GC skew (G − C/G + C. Outwards indicates > 0 and inwards indicates < 0). the dark circles in the middle represent the GC content (outwards indicates greater than the average GC content compared with the whole genome, and inwards indicates the opposite); The outermost circle represents ORFs encoded in the genome, with different colors representing different functions (clockwise arrow indicates the forward reading frame, counterclockwise arrow indicates the reverse reading frame).

To understand more about phage biology, the phage genome was sequenced. Finally, 749606 raw readswith average length of 300 bp was obtained and one contig of 82712bp was assembled after trimming. The average depth of phage contig is 148 after assembly. The termini of the phage genome were predicted by using PhageTerm v1.0.12.1, suggesting that the phage genome has a fixed terminus for packaging and that the other termini may be generated randomly by a headful packaging mechanism. The complete circular genome map of phage vB_VpS_PG28is shown in **Fig 4**. The complete genome sequence of phage vB_VpS_PG28 has a GC content of 48.08%. No tRNA genes were detected in the genome of phage vB_VpS_PG28, indicating that vB_VpS_PG28 depends on the translation machinery of the host.

The genome annotation analysis predicted 114 open reading frames in the complete vB_VpS_PG28 genome **(S1 Table)**, with ATG (110/114), TTG (1/114), and GTG (3/114) serving as start codons and TAA (71/114), TAG (22/114), and TGA (21/114) serving as stop codons. Coding density of open reading frames is 93.828%, covering a total of 77607bp.

### 3.6 Phylogenetic analysis

**Fig 5:**
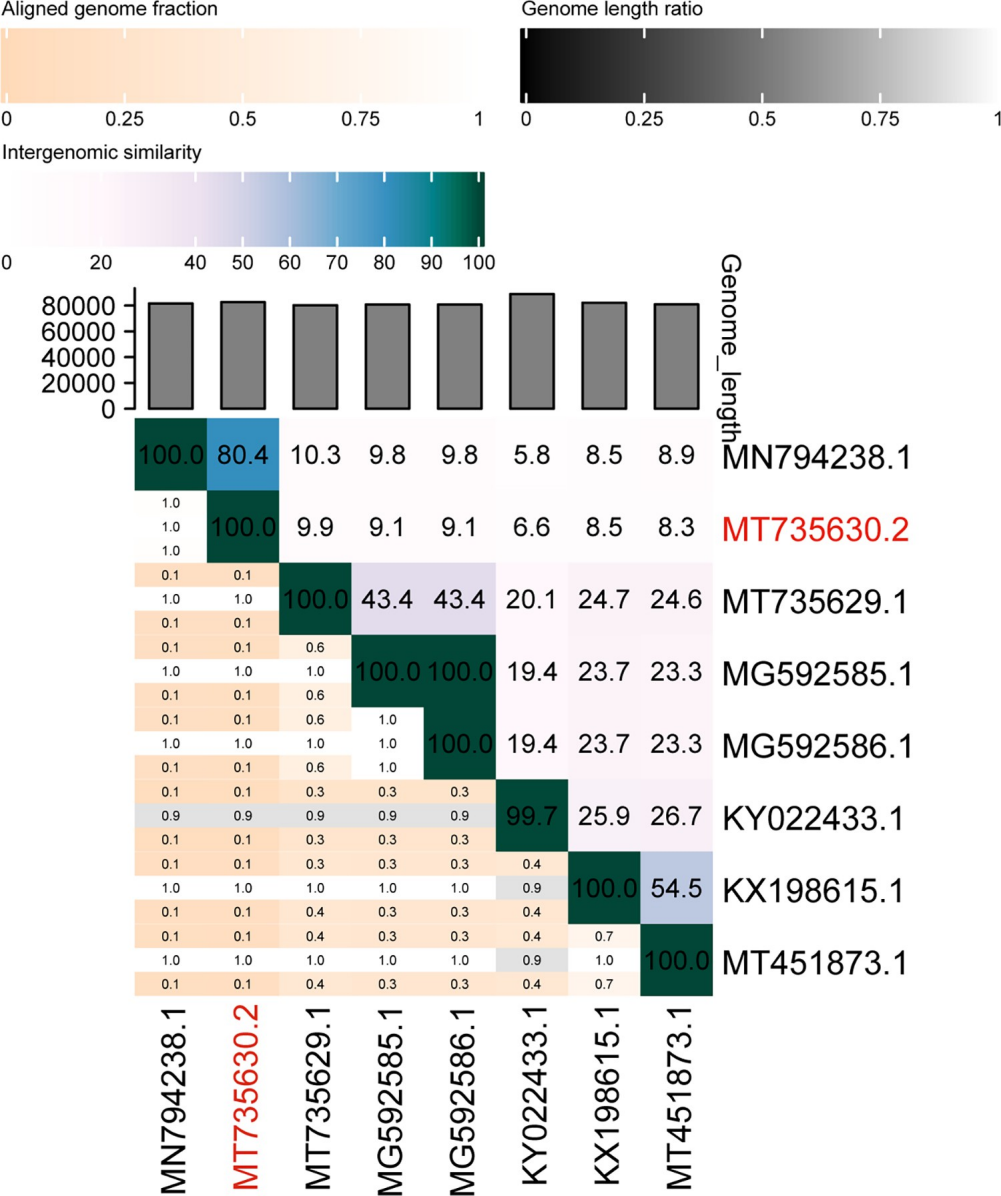
Percent sequence similarity between phages calculated using VIRIDIC. The horizontal and vertical coordinates indicate the corresponding phage GenBank Accession number, and the phage in this study is marked in red font.

To determine the intergenomic similarity between vB_VpS_PG28 and other phages, a heatmap was generated using VIRDIC **(Fig 5)**, resulting that vB_VpS_PG28 has the maximum similarity with *Vibrio* phage VH2_2019 (80.4%), and the homology with other phages were less than 10%. These two phages are sufficient to be classified at the level of a new genus.

**Fig 6:**
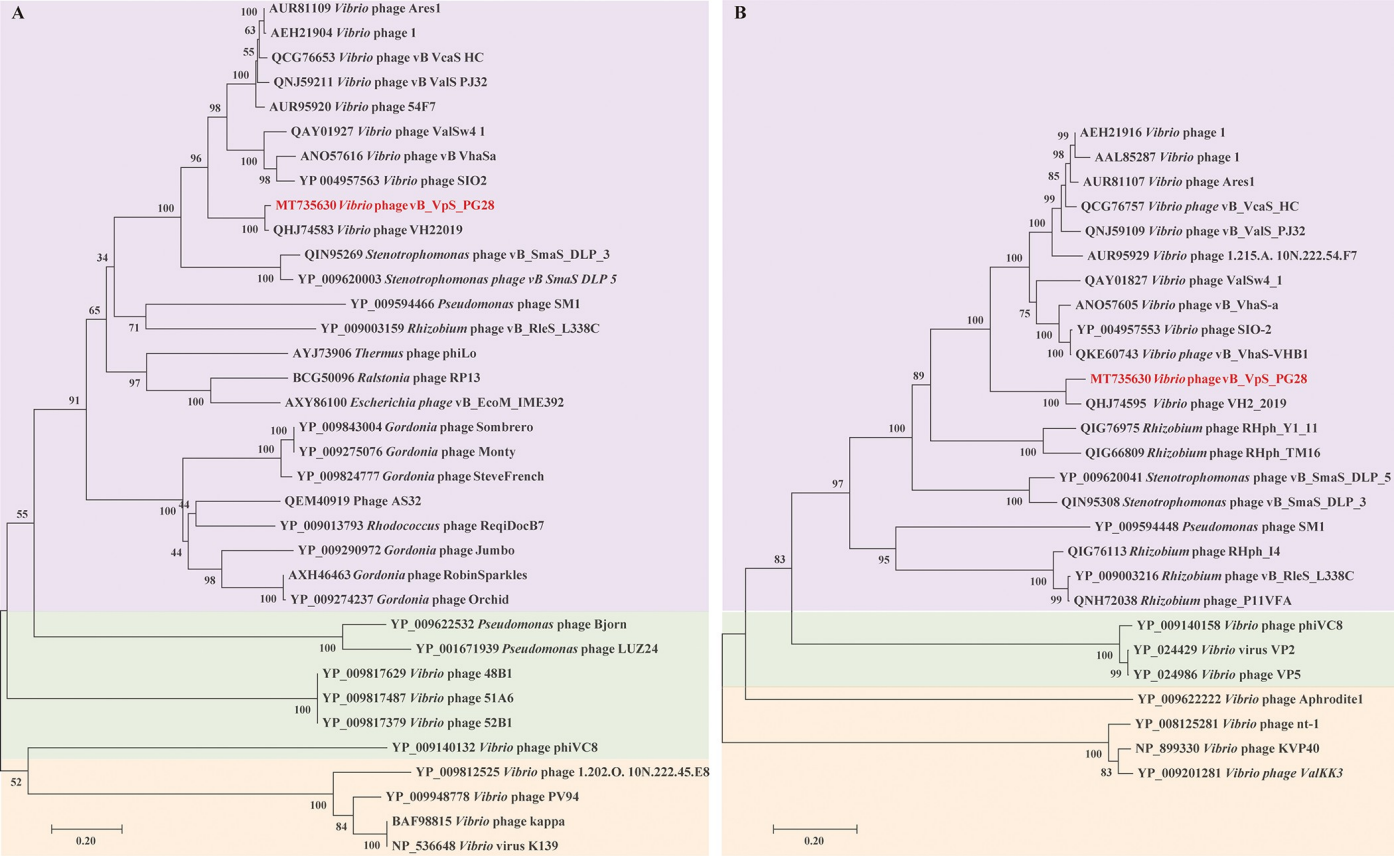
Phylogenetic relationship between selected phage amino acid sequences. **(A)** Tree assembled with terminase large subunit sequences. **(B)** Tree assembled with DNA polymerase sequences. The phages of family Siphoviridae are shown in purple. The phages which belonged to family Podoviridae and Myoviridae are selected as outgroups and shown in green and yellow, respectively. The *Vibrio* phage vB_VpS_PG28 is marked in red.

In the phylogenetic trees based on terminase large subunit and DNA polymerase, vB_VpS_PG28 was clustered with Siphoviridae phages and distant from Myoviridae phages **(Fig 6A and 6B)**. Total number of phages used for this analysis were divided into Podoviridae, Siphoviridae and Myoviridae according to genome data available in the NCBI database. Interestingly, vB_VpS_PG28 was similar to be the members of Myoviridae family by its morphological characteristics.

### 3.7 Comparative analysis

The Bacterial and Archaeal Viruses Subcommittee (BAVS) of the ICTV describes that all species should differ from each other at least 5% of their genome sequence according to a BLASTn search. BLASTn analysis showed that the genome of *Vibrio* phage vB_VpS_PG28 showed high sequence identity (88.81% over 93% query cover) to the genome of *Vibrio* phage VH2_2019(MN794238.1). The genetic kinships between these phages could be related to similarity in their biological properties because the conserved core genes involved in the replication and morphogenesis modules of each genome. Interestingly, these bacteriophages give efficacious results in controlling the infections caused by *Vibrio*, suggesting that phagevB_VpS_PG28 may prove a biological control agent. Complex evolutionary relationships can be predicted between these two phages as both phages were isolated from different territories of the world [40].

**Fig 7:**
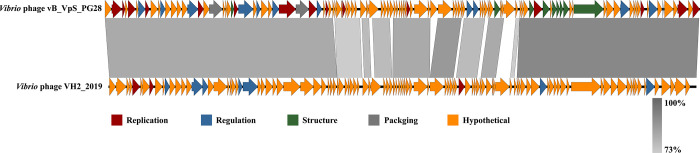
Multiple-sequence alignment of phage genomes. The whole genomes of Vibrio phage vB_VpS_PG28 and Vibrio phage VH2_2019were compared using Easyfig. The grey shading indicates sequence similarities between the genomes.

Genes conservation between these two genomes may demonstrate that the phages possessed ancestral structural genes to sustain their infective capacity to establish infective cycle on bacterial hosts. Oppositely, the tail protein encoded by phage vB_VpS_PG28 show a greater divergence. To confer the host specificity of a phage, tail proteins are involved in host recognition. Moreover, even these two phages share high DNA sequence homology but could show different host specificities. The possible reason behind this, small differences in tail fiber proteins that frequently related to remarkable differences in host ranges and other biological properties. **Fig 7** displays the comparative analysis betweenthese two phage genomes. Both of them have a lot of hypothetical proteins, indicating that their genomes are newly discovered.

Thephage vB_VpS_PG28 genome has a high gene density 1.38 genes per kilobase. Genomic analysis of the phage vB_VpS_PG28 suggests that it is strictly lytic (no lysogenic genes were detected) and does not encode any gene associated with virulence determinants or any immunereactive allergens in their genomes. Therefore, this is the more desirable feature of any phage to use it as a biocontrol agent. However, further testing related to oral toxicity is required to ensure the safe usage of phage.

The genome of phage vB_VpS_PG28 has a comprehensive organization of gene structure that is commonly seen in tailed bacteriophages and every specific structure in this consists of number of genes that have role in similar metabolic pathways including packaging of DNA, morphogenesis structure, replication modules, DNA metabolism and cell lysis.

#### 3.7.1 Structural protein analysis

Structural module of phage vB_VpS_PG28 is located in the central position of the gene sequence. This module mainly included major capsid, head completion, tail, tail tape measure and neck protein.

The module for the head structural components involved ORF88 and ORF90 based on a comparison with other phage head proteins in the NCBI database. Blastp analysis predicted that ORF88 encoded a major capsid protein and exhibited 74% identity to that of *Vibrio* phage vB_VhaS-VHB1. The presumed product of ORF90 showed similarity (36.62% identity) with the head completion protein of *Vibrio* phage1.215.A._10N.222.54.F7.Comparative analysis revealed that major capsid protein encoded by ORF40 showed close relation (74% similarity) with head proteins of *Vibrio* phage.

The tail of phage vB_VpS_PG28 was composed entirely of three proteins (ORF92, ORF93, ORF96) including the tail completion protein, major tail protein, and tail tape measure protein. The product of ORF92 showed 49.30% homology with the tail protein of *Vibrio* phage vB_Vals_PJ32. One putative major tail protein (ORF93) and one putative tail tape measure protein (ORF97) have been predicted in the phage vB_VpS_PG28, which exhibited 61.98% identity to that of *Vibrio* phage vB_Vcas_HC and 42.84% identity to that of *Vibrio* phage vB_Vhas_VHB1, respectively. Furthermore, ORF91 showed 60.78% similarity with *Vibrio* phage vB_Vcas_HC.Phage neck plays a role in the association of the virion head and tail after packaging of viral DNA within the head [41]. Together with gp14, forms a neck at the portal vertex of the head to be ready for the tail attachment.

#### 3.7.2 DNA packaging module

DNA packaging module was also identified in the genome of vB_VpS_PG28 which includes terminase large subunit and portal protein. ORF19 encoded the terminase large subunit which showed 64% identity to *Vibrio* phage 1.215.A._10N.222.54.F7. It is responsible for DNA splicing and packaging. Terminase large subunit may bind and cut specifically near the initiation packaging site [42]. Another important function of this protein is the translocation of DNA powered by ATP [43]. ORF 32 encodes a portal protein with 45.45% similarity with *Vibrio* phage 1.215.A._10N.222.54.F7. It is an important protein in all aspects of bacteriophages including packaging, maturation process and maintain a conserved function. Owing their dynamic role, portal proteins are found variable, and their conformations alters at every assembly stage [44]. As the maturation process is associated with the portal protein, more research is needed to validate this protein as a potent antiviral drug target.

#### 3.7.3 Replication module

The replication module of phage vB_VpS_PG28 is scattered throughout in its genome. It mainly includes DNA helicase(ORF2), DNA binding protein(ORF3), RecA(ORF5), endodeoxyribonuclease(ORF8), DNA polymerase I(ORF31), RNaseH(ORF60) and DNA primase(ORF114).

DNA primase encoded by ORF114 showed 35% similarity with Rhizobium phage (accession no. QIG76855.1). DNA replication has a semi-discontinuous nature, that is why primases are needed for the initiation and lagging strand replication [45]. Furthermore, primase interact with N-terminus of helicase to form a replicator, which plays crucial role in DNA replication, repair, and transcription. Moreover, PSI-BLAST analysis showed that ORF31 of phage vB_VpS_PG28 encoded DNA polymerase, which helps in synthesizing double stranded DNA during replication process. Of note, the genome of phagevB_VpS_PG28 was observed to carry a gene (ORF60) that encodes RNaseH. RNase H enzyme is mostly abundant in the cell’s cytoplasm and nucleus. It contained the essential genetic information and responsible for cleaving the RNA bases and repairing of DNA in RNA–DNA hybridduring replication process. The non-processing of the RNA from RNA/DNA duplex could lead to instability of DNA [46, 47]. Putative protein RecA encoded by ORF5 can be regulated by the function of other proteins and play a role in recombinational DNA repair [48]. Furthermore, ORF8 encodes endo deoxyribonuclease, which is combination of these two proteins, endo nucleases and deoxy ribonucleases. They catalyze the cleavage of the phosphodiester bonds in DNA, which involves in breakage of phage linear single-strand or circular double-stranded DNA molecules [49].

Proteins that bind to DNA are ubiquitous in biology. The ability of these proteins to bind to specific DNA sequences with high affinity is often central to their function, and it is not uncommon for a single mutation to affect the protein ability to bind to the DNA [50].

#### 3.7.4 Lysis module and R-M system

The Lytic gene (ORF11) is adjacent to the replication module. ORF11 was predicted as a lysis protein which shared 95% identity to the N-acetyl-alpha-D-glucosaminyl L-malate deacetylase 1 of *Vibrio* phage VH2_2019. Lysis protein can destroy the cell wall peptidoglycan structure which suggests that the lytic mechanism of phage vB_VpS_PG28is predicted to be accomplished by this protein.

Bacteria protect themselves from the attacks of bacteriophages or any other foreign DNA by using Restriction-modification systems (R-M). Nuclease and methyltransferase enzymes are considered to be R-M systems [51]. The orf33 and orf34 codes ParB-like nuclease domain protein and DNA methyltransferase, respectively. It suggested that host bacteria 6A has the type I restriction-modification system.

## 4 Conclusions

Current study presented, the biological and genomic characteristics of phage vB_VpS_PG28. The results showed that phage vB_VpS_PG28 acts as a promising phage inphage therapy and/or food protection. Various futures were observed advantageous: (I) apparently absence of virulent genes(according to the genomic analysis by VFDB and RESFINDER); (II) Clear plaque formation and absence of genes related to lysogenization, representing the virulence-only type of development; (III) efficient adsorption to host cells; (IV) effective lytic development; and (V) relatively more resistant to different environmental factors (pH & temperature). Overall, based on these properties we propose that further research on vB_VpS_PG28 may provide an avenue to drive its application in food protection on an industrial level.

### 4.1 GenBank accession number

Complete genome sequence of phage vB_VpS_PG28 and 16s were submitted to GenBank under the accession numbers MT735630 and MZ226961, respectively. Raw reads were submitted to NCBI under the SRA accession number SRR14274268.

## Supporting information

S1 Table Annotation of phage vB_VpS_PG28 genes. (DOCX)

S1 Raw image (TIF)
