# Supplementary material for: Characterization and complete genome sequence analysis of a newly isolatedphage against Vibrio parahaemolyticus from sick shrimp in Qingdao, China
Source: PLoS One. 2022 May 4;17(5):e0266683. doi: 10.1371/journal.pone.0266683 (PMC9067683; doi:10.1371/journal.pone.0266683)
Supplement: S1 Table — (DOCX) [file pone.0266683.s001.docx]

Table S1 Annotation of phage vB_VpS_PG28 genes

| ORF no: | START | STOP | F/R | predicted function | Best match | Accession number | E value | cover | identity | MW[KDa] | calc.pI |
| --- | --- | --- | --- | --- | --- | --- | --- | --- | --- | --- | --- |
| 1 | 2 | 898 | F | hypothetical protein | Vibrio phage VH2_2019 | QHJ74565.1 | 0.E+00 | 99% | 90.24% | 32.46 | 5.33 |
| 2 | 950 | 2359 | F | putative DNA helicase | Vibrio phage vB_VcaS_HC | QCG76665.1 | 0.E+00 | 99% | 57% | 53.35 | 9.31 |
| 3 | 2417 | 2824 | F | putative DNA-binding domain protein | Vibrio phage vB_VcaS_HC | QCG76664.1 | 5.E-28 | 95% | 43.61% | 15.63 | 9.54 |
| 4 | 2815 | 3171 | F | hypothetical protein | Vibrio phage VH2_2019 | QHJ74568.1 | 1.E-72 | 100% | 88.14% | 13 | 4.45 |
| 5 | 3219 | 4280 | F | protein RecA | Vibrio phage VH2_2019 | QHJ74569.1 | 0.E+00 | 99% | 97.43% | 29.9 | 5.68 |
| 6 | 4357 | 4641 | F | hypothetical protein | Vibrio phage VH2_2019 | QHJ74570.1 | 9.E-60 | 100% | 93.62% | 10379.84 | 7.97 |
| 7 | 4634 | 5620 | F | rubredoxin-type fold protein | Vibrio phage 1.215.A._10N.222.54.F7 | AUR96018.1 | 8.E-117 | 97% | 54.63% | 36932.66 | 9 |
| 8 | 5617 | 6171 | F | crossover junction endodeoxyribonuclease | Vibrio phage VH2_2019 | QHJ74572.1 | 2.E-125 | 99% | 92.90% | 20731.13 | 5.93 |
| 9 | 6373 | 7353 | F | hypothetical protein | Vibrio phage VH2_2019 | QHJ74573.1 | 0.E+00 | 97% | 89.31% | 35346.98 | 4.93 |
| 10 | 7419 | 7709 | F | hypothetical protein | Vibrio phage VH2_2019 | QHJ74574.1 | 7.E-51 | 100% | 79.17% | 10885.53 | 9.26 |
| 11 | 7711 | 8388 | F | N-acetyl-alpha-D-glucosaminyl  L-malate deacetylase 1 | Vibrio phage VH2_2019 | QHJ74575.1 | 4.E-159 | 100% | 95.11% | 24797.37 | 5.54 |
| 12 | 8388 | 9125 | F | hypothetical protein | Vibrio phage VH2_2019 | QHJ74576.1 | 8.E-154 | 100% | 81.63% | 28429.01 | 8.96 |
| 13 | 9182 | 10033 | F | hypothetical protein | Vibrio phage VH2_2019 | QHJ74577.1 | 0.E+00 | 100% | 95.76% | 32614.12 | 6.15 |
| 14 | 10014 | 10634 | F | hypothetical protein | Vibrio phage VH2_2019 | QHJ74578.1 | 2.E-138 | 99% | 92.16% | 22842.92 | 5.36 |
| 15 | 10714 | 11460 | F | hypothetical protein | Vibrio phage VH2_2019 | QHJ74579.1 | 1.E-153 | 100% | 83.47% | 27966.97 | 5.63 |
| 16 | 11465 | 12976 | F | glycosyltransferase | Vibrio phage VH2_2019 | QHJ74580.1 | 0.E+00 | 100% | 93.24% | 57875.17 | 6.12 |
| 17 | 12951 | 13718 | F | nucleoid occlusion protein | Vibrio phage VH2_2019 | QHJ74581.1 | 6.E-174 | 100% | 91.37% | 28812.86 | 5.54 |
| 18 | 13708 | 14514 | F | coil containing protein | Vibrio phage 1.215.A._10N.222.54.F7 | AUR95919.1 | 6.E-71 | 98% | 64.55% | 29658.82 | 9.26 |
| 19 | 14501 | 16354 | F | terminase large subunit | Vibrio phage 1.215.A._10N.222.54.F7 | AUR95920.1 | 0.E+00 | 98% | 64.55% | 69848.75 | 5.55 |
| 20 | 16405 | 16647 | F | hypothetical protein | Vibrio phage VH2_2019 | QHJ74584.1 | 3.E-45 | 100% | 87.5% | 8965.23 | 6.16 |
| 21 | 16757 | 16984 | F | hypothetical protein | Vibrio phage VH2_2019 | QHJ74585.1 | 8.E-41 | 100% | 86.67% | 8876.29 | 9.69 |
| 22 | 16984 | 17535 | F | hypothetical protein | Vibrio phage VH2_2019 | QHJ74586.1 | 4.E-122 | 99% | 91.76% | 20524.56 | 7.8 |
| 23 | 17546 | 17938 | F | coil containing protein | Vibrio phage vB_ValS_PJ32 | QNJ59215.1 | 6.E-10 | 74% | 38.14% | 14546.96 | 9.95 |
| 24 | 17935 | 18486 | F | deoxycytidine triphosphate deaminase | Vibrio phage VH2_2019 | QHJ74588.1 | 2.E-123 | 100% | 89.07% | 20582.91 | 6.74 |
| 25 | 18560 | 20617 | F | pyruvate/phosphate dikinase | Vibrio phage VH2_2019 | QHJ74589.1 | 0.E+00 | 100% | 81.61% | 75161.97 | 5.07 |
| 26 | 20620 | 21093 | F | hypothetical protein | Vibrio phage VH2_2019 | QHJ74590.1 | 3.E-93 | 100% | 84.71% | 17309.32 | 4.71 |
| 27 | 21080 | 21637 | F | putative protein-tyrosine phosphatase | Vibrio phage vB_VcaS_HC | QCG76646.1 | 4.E-31 | 89% | 38.55% | 20640.66 | 6.43 |
| 28 | 21724 | 22704 | F | hypothetical protein | Vibrio phage VH2_2019 | QHJ74592.1 | 1.E-172 | 100% | 73.93% | 36531.56 | 6.39 |
| 29 | 22701 | 23240 | F | hypothetical protein | Vibrio phage VH2_2019 | QHJ74593.1 | 4.E-101 | 100% | 77.65% | 20327.25 | 8.72 |
| 30 | 23292 | 24188 | F | transporter | Vibrio phage vB_ValS_PJ32 | QNJ59108.1 | 3.E-24 | 94% | 29.82% | 32200.8 | 5.68 |
| 31 | 24198 | 26537 | F | DNA polymerase I | Vibrio phage vB_VhaS-VHB1 | QKE60743.1 | 0.E+00 | 100% | 60.46% | 87975.26 | 6.22 |
| 32 | 26539 | 28359 | F | gene transfer agent portal protein | Vibrio phage 1.215.A._10N.222.54.F7 | AUR95930.1 | 0.E+00 | 98% | 45.45% | 67267.32 | 4.96 |
| 33 | 28361 | 29455 | F | ParB-like nuclease domain protein | Stenotrophomonas phage vB_SmaS_DLP_5 | YP_009619897.1 | 8.E-20 | 74% | 26.94% | 40252.78 | 4.74 |
| 34 | 29467 | 30171 | F | DNA methyltransferase | Vibrio phage vB_VcaS_HC | QCG76754.1 | 7.E-86 | 86% | 64.04% | 27047.97 | 8.45 |
| 35 | 30364 | 30615 | F | hypothetical protein | Vibrio phage VH2_2019 | QHJ74599.1 | 2.E-33 | 98% | 71.95% | 36345.72 | 8.93 |
| 36 | 30700 | 31092 | F | TMhelix containing protein | Vibrio phage vB_ValS_PJ32 | QNJ59113.1 | 1.E-16 | 86% | 37.17% | 14806.01 | 5.52 |
| 37 | 31103 | 31522 | F | hypothetical protein | Vibrio phage VH2_2019 | QHJ74601.1 | 2.E-81 | 100% | 84.89% | 15010.42 | 8.27 |
| 38 | 31651 | 31920 | F | not hits | - | - | - | - | - | 10030.71 | 9.05 |
| 39 | 31932 | 32168 | F | hypothetical protein | Vibrio phage VH2_2019 | QHJ74602.1 | 2.E-45 | 100% | 92.31% | 8863.3 | 9.72 |
| 40 | 32170 | 32862 | F | hypothetical protein | Vibrio phage VH2_2019 | QHJ74603.1 | 3.E-143 | 100% | 83.48% | 25931.56 | 5.59 |
| 41 | 32852 | 33004 | F | - | - | - | - | - | - | 5640.93 | 10.02 |
| 42 | 33004 | 33468 | F | putative DNA polymerase I | Vibrio phage vB_ValS_PJ32 | QNJ59115.1 | 2.E-20 | 79% | 38.89% | 17258.5 | 6.38 |
| 43 | 33461 | 33709 | F | hypothetical protein | Vibrio phage VH2_2019 | QHJ74605.1 | 1.E-30 | 97% | 62.5% | 9627.38 | 7.67 |
| 44 | 33716 | 34234 | F | hypothetical protein | Vibrio phage VH2_2019 | QHJ74606.1 | 8.E-74 | 100% | 59.88% | 20141.49 | 9.62 |
| 45 | 34246 | 34746 | F | hypothetical protein | Vibrio phage VH2_2019 | QHJ74607.1 | 2.E-81 | 99% | 69.7% | 19192.15 | 9.24 |
| 46 | 34743 | 35039 | F | hypothetical protein | Vibrio phage VH2_2019 | QHJ74608.1 | 2.E-42 | 100% | 69.39% | 10903.45 | 5.34 |
| 47 | 35513 | 36343 | F | hypothetical protein | Vibrio phage VH2_2019 | QHJ74609.1 | 1.E-160 | 100% | 77.17% | 31969.41 | 9.48 |
| 48 | 36343 | 36633 | F | hypothetical protein VH22019_00046 | Vibrio phage VH2_2019 | QHJ74610.1 | 8.E-33 | 98% | 72.63% | 11158.14 | 9.36 |
| 49 | 36630 | 38033 | F | hypothetical protein VH22019_00047 | Vibrio phage VH2_2019 | QHJ74611.1 | 0.E+00 | 100% | 58.64% | 53269.93 | 8.57 |
| 50 | 38374 | 38745 | F | hypothetical protein VH22019_00048 | Vibrio phage VH2_2019 | QHJ74612.1 | 6.E-54 | 100% | 66.67% | 14179.07 | 5.14 |
| 51 | 38766 | 39209 | F | hypothetical protein VH22019_00049 | Vibrio phage VH2_2019 | QHJ74613.1 | 2.E-85 | 100% | 83.22% | 16822.7 | 4.71 |
| 52 | 39223 | 39621 | F | hypothetical protein VH22019_00050 | Vibrio phage VH2_2019 | QHJ74614.1 | 5.E-68 | 96% | 75.00% | 15334.67 | 9.24 |
| 53 | 39621 | 40136 | F | hypothetical protein | Peribacillus asahii | WP_127760722.1 | 2.E-03 | 29% | 41.18% | 19782.57 | 9.81 |
| 54 | 40154 | 40459 | F | hypothetical protein VH22019_00051 | Vibrio phage VH2_2019 | QHJ74615.1 | 5.E-35 | 100% | 62.38% | 11342.87 | 4.80 |
| 55 | 40512 | 40745 | F | hypothetical protein VH22019_00052 | Vibrio phage VH2_2019 | QHJ74616.1 | 8.E-29 | 100% | 68.83% | 8490.75 | 4.74 |
| 56 | 40881 | 41135 | F | hypothetical protein VH22019_00053 | Vibrio phage VH2_2019 | QHJ74617.1 | 8.E-43 | 100% | 82.14% | 9945.39 | 8 |
| 57 | 41183 | 41455 | F | hypothetical protein VH22019_00054 | Vibrio phage VH2_2019 | QHJ74618.1 | 2.E-51 | 100% | 87.78% | 10473.53 | 4.42 |
| 58 | 41475 | 41678 | F | hypothetical protein VH22019_00055 | Vibrio phage VH2_2019 | QHJ74619.1 | 1.E-40 | 100% | 97.01% | 7672.93 | 9.78 |
| 59 | 41692 | 41940 | F | hypothetical protein VH22019_00056 | Vibrio phage VH2_2019 | QHJ74620.1 | 1.E-40 | 100% | 81.71% | 9203.31 | 4.8 |
| 60 | 41945 | 42430 | F | RNase H | Vibrio virus vB_VspP_SBP1 | AZU99699.1 | 1.E-29 | 98% | 45.34% | 18083.83 | 9.51 |
| 61 | 42423 | 42827 | F | hypothetical protein VH22019_00058 | Vibrio phage VH2_2019 | QHJ74622.1 | 2.E-86 | 100% | 94.03% | 15270.44 | 9.49 |
| 62 | 43008 | 44900 | F | hypothetical protein VH22019_00059 | Vibrio phage VH2_2019 | QHJ74623.1 | 0.E+00 | 100% | 80.00% | 68152.14 | 4.57 |
| 63 | 44911 | 45147 | F | hypothetical protein VH22019_00060 | Vibrio phage VH2_2019 | QHJ74624.1 | 3.E-39 | 100% | 83.33% | 8558.83 | 5.59 |
| 64 | 45249 | 46199 | F | hypothetical protein vBVcaS_HC063 | Vibrio phage vB_VcaS_HC | QCG76707.1 | 2.E-11 | 98% | 27.69% | 33461.67 | 4.64 |
| 65 | 46317 | 48230 | F | hypothetical protein VH22019_00061 | Vibrio phage VH2_2019 | QHJ74625.1 | 0.E+00 | 100% | 92.15% | 69208.45 | 4.74 |
| 66 | 48375 | 48728 | F | hypothetical protein VH22019_00062 | Vibrio phage VH2_2019 | QHJ74626.1 | 2.E-59 | 100% | 74.36% | 13613.6 | 7.73 |
| 67 | 48742 | 49074 | F | hypothetical protein VH22019_00063 | Vibrio phage VH2_2019 | QHJ74627.1 | 1.E-60 | 100% | 85.45% | 12297.3 | 5.21 |
| 68 | 49076 | 49477 | F | hypothetical protein VH22019_00064 | Vibrio phage VH2_2019 | QHJ74628.1 | 2.E-70 | 99% | 76.52% | 14744.8 | 5.3 |
| 69 | 49479 | 49727 | F | hypothetical protein VH22019_00065 | Vibrio phage VH2_2019 | QHJ74629.1 | 1.E-26 | 100% | 58.54% | 9039.51 | 7.84 |
| 70 | 49724 | 50083 | F | hypothetical protein VH22019_00066 | Vibrio phage VH2_2019 | QHJ74630.1 | 2.E-39 | 100% | 54.92% | 13106.12 | 9.75 |
| 71 | 50304 | 51143 | F | polyamine aminopropyltransferase | Vibrio phage VH2_2019 | QHJ74631.1 | 3.E-169 | 99% | 79.86% | 30860.51 | 4.96 |
| 72 | 51203 | 51964 | F | radical SAM protein | Vibrio phage VH2_2019 | WP_011939412.1 | 2.E-29 | 76% | 37.21% | 27812.28 | 4.52 |
| 73 | 52139 | 52441 | F | hypothetical protein VH22019_00069 | Vibrio phage VH2_2019 | QHJ74633.1 | 3.E-60 | 100% | 90.00% | 11102.09 | 6.06 |
| 74 | 52454 | 52732 | F | hypothetical protein VH22019_00070 | Vibrio phage VH2_2019 | QHJ74634.1 | 3.E-50 | 100% | 84.78% | 10426.38 | 10.29 |
| 75 | 52744 | 53244 | F | hypothetical protein VH22019_00071 | Vibrio phage VH2_2019 | QHJ74635.1 | 4.E-58 | 100% | 59.41% | 18213.83 | 4.43 |
| 76 | 53241 | 53474 | F | hypothetical protein VH22019_00072 | Vibrio phage VH2_2019 | QHJ74636.1 | 2.E-21 | 97% | 58.67% | 8781.51 | 3.62 |
| 77 | 53474 | 54007 | F | hypothetical protein VH22019_00073 | Vibrio phage VH2_2019 | QHJ74637.1 | 8.E-112 | 100% | 85.88% | 20578.36 | 5.9 |
| 78 | 54157 | 54930 | F | coil containing protein | Vibrio phage 1.215.A._10N.222.54.F7 | AUR95984.1 | 2.E-78 | 100% | 45.00% | 29630.31 | 9.4 |
| 79 | 54976 | 55173 | F | hypothetical protein VH22019_00075 | Vibrio phage VH2_2019 | QHJ74639.1 | 2.E-39 | 100% | 98.46% | 7622.67 | 7.89 |
| 80 | 55160 | 55405 | F | hypothetical protein VH22019_00076 | Vibrio phage VH2_2019 | QHJ74640.1 | 1.E-49 | 98% | 95.00% | 9026.11 | 6.81 |
| 81 | 55405 | 57009 | F | hypothetical protein VH22019_00077 | Vibrio phage VH2_2019 | QHJ74641.1 | 2.E-02 | 5% | 70.00% | 60661.3 | 5.48 |
| 82 | 57074 | 57412 | F | hypothetical protein VH22019_00078 | Vibrio phage VH2_2019 | QHJ74642.1 | 2.E-47 | 100% | 68.75% | 11589.04 | 4.64 |
| 83 | 57668 | 57898 | F | hypothetical protein VH22019_00080 | Vibrio phage VH2_2019 | QHJ74644.1 | 1.E-39 | 100% | 84.21% | 8785.8 | 4.55 |
| 84 | 57900 | 58211 | F | hypothetical protein VH22019_00081 | Vibrio phage VH2_2019 | QHJ74645.1 | 4.E-66 | 100% | 97.09% | 11932.74 | 4.88 |
| 85 | 58208 | 58867 | F | hypothetical protein VH22019_00082 | Vibrio phage VH2_2019 | QHJ74646.1 | 6.E-154 | 100% | 93.61% | 25018.46 | 5.51 |
| 86 | 59002 | 59631 | F | XkdF | Vibrio phage vB_VcaS_HC | QCG76691.1 | 3.E-80 | 96% | 60.59% | 23503.85 | 4.38 |
| 87 | 59638 | 60810 | F | transport and binding protein | Vibrio phage vB_VhaS-VHB1 | QKE60696.1 | 2.E-47 | 96% | 35.84% | 41906.66 | 4.33 |
| 88 | 60878 | 61831 | F | major capsid protein | Vibrio phage vB_VhaS-VHB1 | QKE60697.1 | 4.E-169 | 99% | 74.29% | 35221.18 | 5.40 |
| 89 | 61905 | 62174 | F | hypothetical protein VH22019_00086 | Vibrio phage VH2_2019 | QHJ74650.1 | 1.E-34 | 100% | 68.89% | 10043.33 | 5.14 |
| 90 | 62184 | 62789 | F | head completion adaptor | Vibrio phage 1.215.A._10N.222.54.F7 | AUR95991.1 | 2.E-38 | 100% | 36.62% | 22502.2 | 5.02 |
| 91 | 62786 | 63247 | F | neck protein | Vibrio phage vB_VcaS_HC | QCG76686.1 | 1.E-61 | 98% | 60.78% | 17060.31 | 6.73 |
| 92 | 63244 | 63681 | F | tail-completion protein | Vibrio phage vB_ValS_PJ32 | QNJ59179.1 | 3.E-41 | 95% | 49.30% | 16133.32 | 5.17 |
| 93 | 63739 | 64512 | F | major tail protein | Vibrio phage vB_VcaS_HC | QCG76684.1 | 7.E-107 | 99% | 61.98% | 27543.74 | 4.69 |
| 94 | 64594 | 64995 | F | hypothetical protein VH22019_00091 | Vibrio phage VH2_2019 | QHJ74655.1 | 2.E-72 | 100% | 90.23% | 14520.86 | 5 |
| 95 | 65016 | 65198 | F | - | - | - | - | - | - | 6901.99 | 6.71 |
| 96 | 65202 | 69332 | F | tail tape measure protein | Vibrio phage vB_VhaS-VHB1 | QKE60705.1 | 0.E+00 | 99% | 42.84% | 149698.2 | 5.17 |
| 97 | 69332 | 69718 | F | hypothetical protein VH22019_00093 | Vibrio phage VH2_2019 | QHJ74657.1 | 7.E-87 | 100% | 100.00% | 13646.68 | 4.74 |
| 98 | 69729 | 70724 | F | hypothetical protein VH22019_00094 | Vibrio phage VH2_2019 | QHJ74658.1 | 0.E+00 | 100% | 98.79% | 35416.73 | 4.91 |
| 99 | 70736 | 71653 | F | hypothetical protein VH22019_00095 | Vibrio phage VH2_2019 | QHJ74659.1 | 0.E+00 | 100% | 96.72% | 34003.67 | 4.37 |
| 100 | 71661 | 72953 | F | LamG domain-containing protein | candidate division KSB1 bacterium | MBI5058973.1 | 6.E-33 | 80% | 28% | 47220.81 | 4.96 |
| 101 | 72953 | 73489 | F | hypothetical protein VH22019_00097 | Vibrio phage VH2_2019 | QHJ74661.1 | 8.E-123 | 100% | 96.63% | 19304.59 | 4.46 |
| 102 | 73489 | 73794 | F | hypothetical protein VH22019_00098 | Vibrio phage VH2_2019 | QHJ74662.1 | 1.E-63 | 100% | 99.01% | 10942.71 | 5.13 |
| 103 | 73791 | 74018 | F | hypothetical protein VH22019_00099 | Vibrio phage VH2_2019 | QHJ74663.1 | 5.E-44 | 100% | 98.67% | 8228.52 | 9.68 |
| 104 | 74015 | 74482 | F | hypothetical protein VH22019_000100 | Vibrio phage VH2_2019 | QHJ74664.1 | 1.E-104 | 100% | 92.26% | 17339.88 | 8.8 |
| 105 | 74479 | 75015 | F | TMhelix containing protein | Vibrio phage 1.215.A._10N.222.54.F7 | AUR96005.1 | 2.E-46 | 95% | 45.03% | 19637.69 | 6.08 |
| 106 | 75377 | 75625 | F | hypothetical protein VH22019_000102 | Vibrio phage VH2_2019 | QHJ74666.1 | 5.E-31 | 100% | 63.41% | 9555.51 | 4.22 |
| 107 | 75635 | 76825 | F | ATP-dependent zinc metalloprotease | Vibrio phage VH2_2019 | QHJ74667.1 | 0.E+00 | 99% | 92.41% | 44190.69 | 4.6 |
| 108 | 76827 | 77450 | F | hypothetical protein VH22019_000104 | Vibrio phage VH2_2019 | QHJ74668.1 | 1.E-108 | 99% | 71.84% | 23043.42 | 5.56 |
| 109 | 77543 | 77635 | F | Gfo/Idh/MocA family oxidoreductase | Rhodobacteraceae bacterium | TQS69949.1 | 2.E+01 | 60% | 72.22% | 3183.76 | 9.84 |
| 110 | 77780 | 79024 | F | hypothetical protein VH22019_000105 | Vibrio phage VH2_2019 | QHJ74669.1 | 0.E+00 | 100% | 85.75% | 46354.7 | 9.38 |
| 111 | 79024 | 79608 | F | hypothetical protein VH22019_000106 | Vibrio phage VH2_2019 | QHJ74670.1 | 1.E-129 | 100% | 89.69% | 22848.41 | 7.02 |
| 112 | 79608 | 81038 | F | DnaB-like DNA helicase | Vibrio phage 1.215.A._10N.222.54.F7 | AUR96009.1 | 6.E-141 | 98% | 46.32% | 53556.86 | 5.16 |
| 113 | 81093 | 81725 | F | hypothetical protein VH22019_000108 | Vibrio phage VH2_2019 | QHJ74672.1 | 3.E-94 | 100% | 75.83% | 22332.89 | 9.68 |
| 114 | 81718 | 82704 | F | DNA primase protein | Rhizobium phage RHph_Y1_11 | QIG76855.1 | 7.E-55 | 93% | 35.39% | 37386.97 | 5.87 |
