# Supplementary figures and images for: Characterization and complete genome sequence analysis of a newly isolatedphage against Vibrio parahaemolyticus from sick shrimp in Qingdao, China
Source: PLoS One. 2022 May 4;17(5):e0266683. doi: 10.1371/journal.pone.0266683 (PMC9067683; doi:10.1371/journal.pone.0266683)

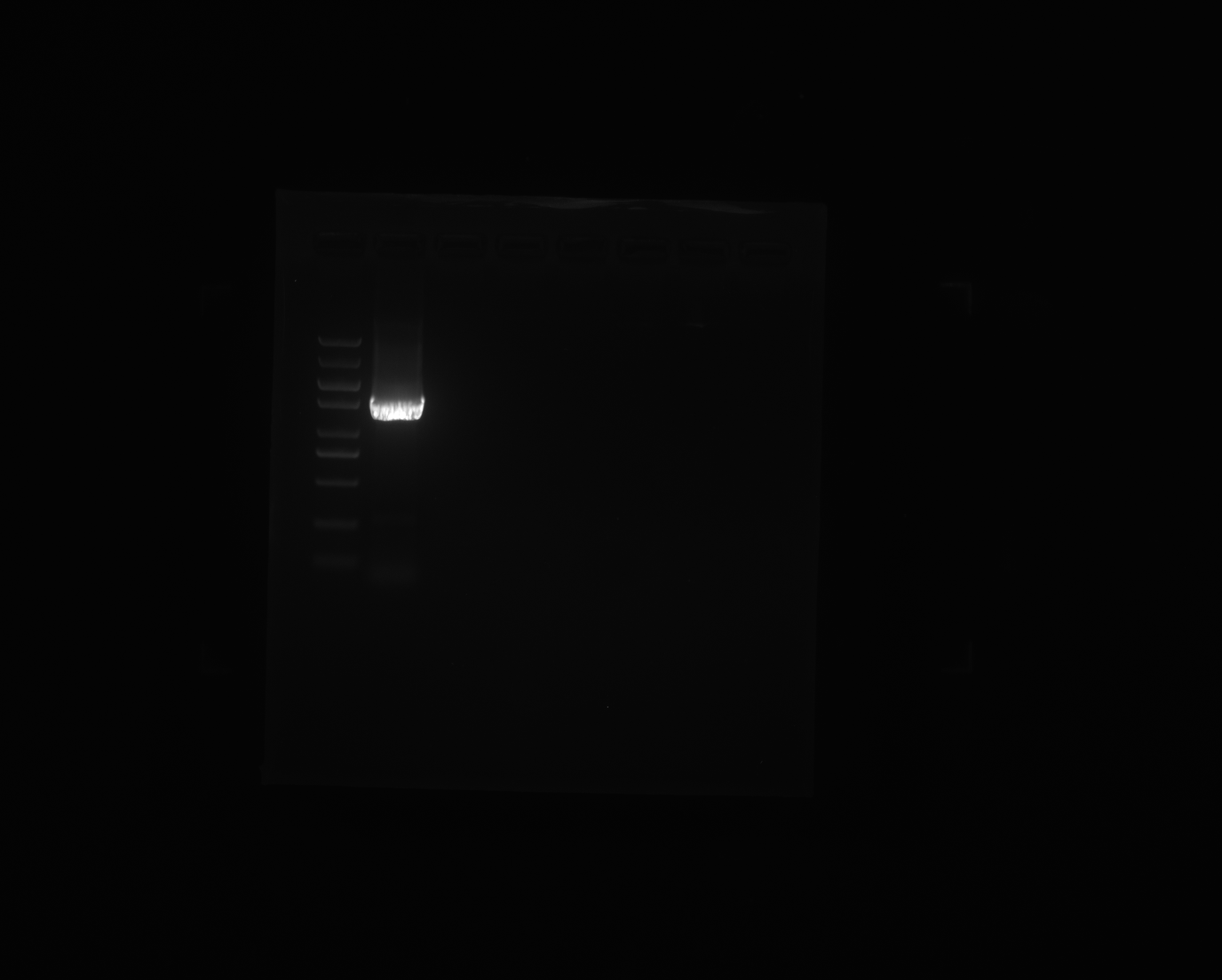

Supplement: S1 Raw image — (TIF) [file pone.0266683.s002.Tif]
